# Chitosan-covered tamponade for the treatment of postpartum hemorrhage: a registry-based cohort study assessing outcomes and risk factors for treatment failure

**DOI:** 10.1186/s12884-025-07236-5

**Published:** 2025-02-05

**Authors:** Clara Leichtle, Annette Aigner, Carolin Biele, Paulina Hermann, Teresa Dangli, Charlotte Waldner, Thorsten Braun, Wolfgang Henrich, Anna Maria Dückelmann

**Affiliations:** 1 Department of Obstetrics, Charité - Universitätsmedizin Berlin, Corporate Member of Freie Universität Berlin and Humboldt Universität zu Berlin, Berlin, Germany; 2 Institute of Biometry and Clinical Epidemiology, Charité - Universitätsmedizin Berlin, Corporate Member of Freie Universität Berlin and Humboldt Universität zu Berlin, Berlin, Germany; 3 Center for Stroke Research Berlin, Charité - Universitätsmedizin Berlin, Corporate Member of Freie Universität Berlin, Humboldt Universität zu Berlin, Berlin, Germany

**Keywords:** Postpartum hemorrhage (PPH), Chitosan-covered gauze, Treatment failure, Uterine packing, Placenta previa

## Abstract

**Background:**

Postpartum hemorrhage (PPH) is one of the leading causes of maternal morbidity and mortality worldwide. Intrauterine hemostatic devices are recommended when PPH does not respond to medical treatment. The objective of this study was to assess the factors leading to unsuccessful intrauterine therapy with a chitosan-covered tamponade (CT) for the treatment of PPH and to evaluate clinical outcomes based on real-world data.

**Methods:**

This registry-based cohort study included all women treated with CT for PPH between January 2017 and June 2022 at a university clinic’s perinatal department. The endpoint was defined as the failure of CT, indicated by the requirement of further invasive procedures for ongoing hemorrhage after CT application. Medical records were reviewed and binary logistic regressions used to evaluate delivery mode, placenta previa, and placenta accreta spectrum as potential risk factors for CT treatment failure.

**Results:**

The cohort consisted of 230 women, with successful CT treatment in 91.3%. The success rate for mild PPH was 100.0%, for moderate 95.5%, and for severe 84.2%. Five hysterectomies were performed in total. Placenta previa in cesarean sections was identified as the primary risk factor for CT treatment failure, increasing the odds about 7.5-fold (Odds Ratio: 7.48; 95% CI: 1.87–33.15) compared to cesarean sections without placenta previa. Furthermore, delays in CT insertion may also contribute to treatment failure.

**Conclusion:**

CT serves as an intrauterine treatment for medically intractable PPH. Placenta previa significantly increases the risk of CT treatment failure in cesarean sections. Obstetricians should be particularly vigilant in managing patients with placenta previa and consider early use of CT or a combination of procedures.

**Trial registration:**

This study was approved by the local Ethics Committee on 11/10/2021 (EA4/231/21).

## Background

Postpartum hemorrhage (PPH) is a sudden and often unpredictable emergency that remains a substantial obstetric challenge, contributing significantly to maternal morbidity and mortality worldwide [1–4]. Affecting approximately 5% of women during childbirth, PPH is the leading cause of maternal mortality worldwide, accounting for approximately 25% of all maternal deaths [5, 6]. Thus, successful management is of utmost importance.

In recent years, new non-balloon intrauterine PPH management tools have emerged on the market [7–9], the most promising of which are the Celox^®^ gauze, a hemostatic chitosan-covered tamponade (CT), and the Jada^®^ System, a vacuum-induced hemorrhage control device [10, 11]. Chitosan functions as a hemostatic agent through a distinct mechanism that does not rely on the typical clotting cascade. Upon contact with blood, the CT forms a gel-like matrix and promotes efficient blood coagulation through electrostatic interactions between chitosan and the cell membranes of erythrocytes, even in the presence of heparin [12–14]. CT was first used to treat PPH in 2012 [15], it is CE-marked and received approval from the European Medicines Agency in 2022.

When introducing a new device in sensitive areas such as obstetrics, sufficient safety and at least adequate efficiency are mandatory for implementation. CT has no relevant side effects in the short and long term and requires minimal training for correct use, with success rates of 91–95% and significant reductions in hysterectomies by 50–75% [11, 16–19], though not based on RCT evidence. Successful treatment of PPH consists of controlled bleeding after application of the treatment without further interventions. Recently, our study group retrospectively investigated the effect of CT on the need for further measures in PPH, comparing it to medical therapy alone and intrauterine balloon tamponade in a cohort of 666 women [20]. The success rate with CT application was 91%, whereas the success rate with balloon management was 84%. In addition, the group who received CT had a lower hysterectomy rate (0/85) than the group treated with balloon tamponade (4/51, 8%).

As the use of CT becomes more widespread in clinical practice, understanding its handling and limitations is crucial to making informed decisions and refining PPH management strategies. Currently, data are lacking on the risk factors for CT treatment failure, which are needed to communicate the limitations of the method and to facilitate recommendations on the appropriate time of use. Our clinic has substantial experience with CT, having used it since 2016. In the present study, we comprehensively analyzed the efficacy of CT in the management of PPH and identified specific factors associated with failure of CT therapy. Based on prospectively and retrospectively collected real-world data from a large cohort of women, the findings aim to enhance recommendations for the appropriate use of CT in PPH management.

## Methods

### Study design and patient selection

The registry on which the current study is based includes all women who give birth at the perinatal department of a university hospital with more than 5,000 deliveries per year and receive CT for PPH. The recruitment started on January 1, 2017, and this study’s sample includes all patients enrolled by June 6, 2022. Exclusion criteria are maternal age < 17 years, the use of CT only for vaginal lacerations, and the performance of other interventions simultaneously or prior to CT, such as a balloon tamponade, artery ligation, embolization, or compression sutures. The patients’ demographic and epidemiological data are collected, as well as detailed information on labor and delivery, blood loss, infection parameters, postpartum course, and child data, by retrospectively reviewing medical records in our data systems (SAP [Walldorf, Germany] and Viewpoint by GE [Solingen, Germany]).

The definition of PPH is based on the guidelines of the Royal College of Obstetricians and Gynecologists (RCOG) [21], which defines PPH as blood loss ≥ 500 mL following vaginal delivery or caesarean section within 24 h (primary PPH) or 12 weeks (secondary PPH) of delivery. Blood loss is estimated using a combination of collection bag volume and the weight of used pads. PPH is categorized by the amount of blood loss into mild (500–1000 mL), moderate (> 1000–2000 mL), or severe (> 2000 mL).

### Treatment routine

The management of PPH according to local guidelines consisted of routine intravenous injection of 3 IU oxytocin or Carbetocin after birth, placement of a graduated collector bag at the start of bleeding, bimanual uterine massage, continuous administration of oxytocin, suturing of lacerations, and removal of the placenta 30 min after birth if not expelled [22]. If bleeding persisted, tranexamic acid and additional uterotonic agents (e.g., misoprostol or sulprostone) were administered. If these measures failed to control the bleeding, the next step was intrauterine therapy. The decision to use CT was made by the obstetrician in charge. In the whole registry we used the Celox^®^ tamponade from Medtrade Products, and each patient received at least one gauze (3 m long, 7.6 cm wide). Depending on the mode of delivery, the CT was inserted either transvaginally in case of dilated cervix or transabdominally in primary cesarean sections, with one end of the tamponade passed through the cervix for subsequent removal after a maximum of 24 h by pulling the end of the gauze left in the vagina, also in cases with undilated cervix. Please refer to the published literature for further details on handling [18].

### Definition of endpoints

The outcome of interest was the failure of treatment with CT. Failure was defined as persistent bleeding more than 5 min after application and requirement of further intervention. The decision on further interventions was made individually according to the hemodynamic deterioration. Treatment options included the intrauterine application of a balloon tamponade, compression sutures, artery ligation, radiographic embolization, and consecutive hysterectomy.

### Statistical analysis

Descriptively, categorical variables are presented as absolute and relative frequencies, and continuous variables as medians with interquartile ranges (IQR), stratified by the endpoint. The temporal trend in frequency of CT failure and placenta previa cases is presented along with 95% Clopper-Pearson confidence intervals (CI). Based on clinical relevance, binary logistic regression models were used to quantify the association between CT failure and placenta accreta spectrum (PAS), a placenta previa (along with a caesarean section) and the performance of caesarean section for a different indication. To derive adjusted effect estimates, maternal age, multiple pregnancies, neonatal weight, and body mass index (BMI) were considered as confounders. Results are presented as odds ratio (OR) estimates with 95% CIs. Analyses were performed using IBM SPSS Statistics (version 28.0.1.0) and R [23], just as the additional R package tidyverse [24].

## Results

**Fig. 1:**
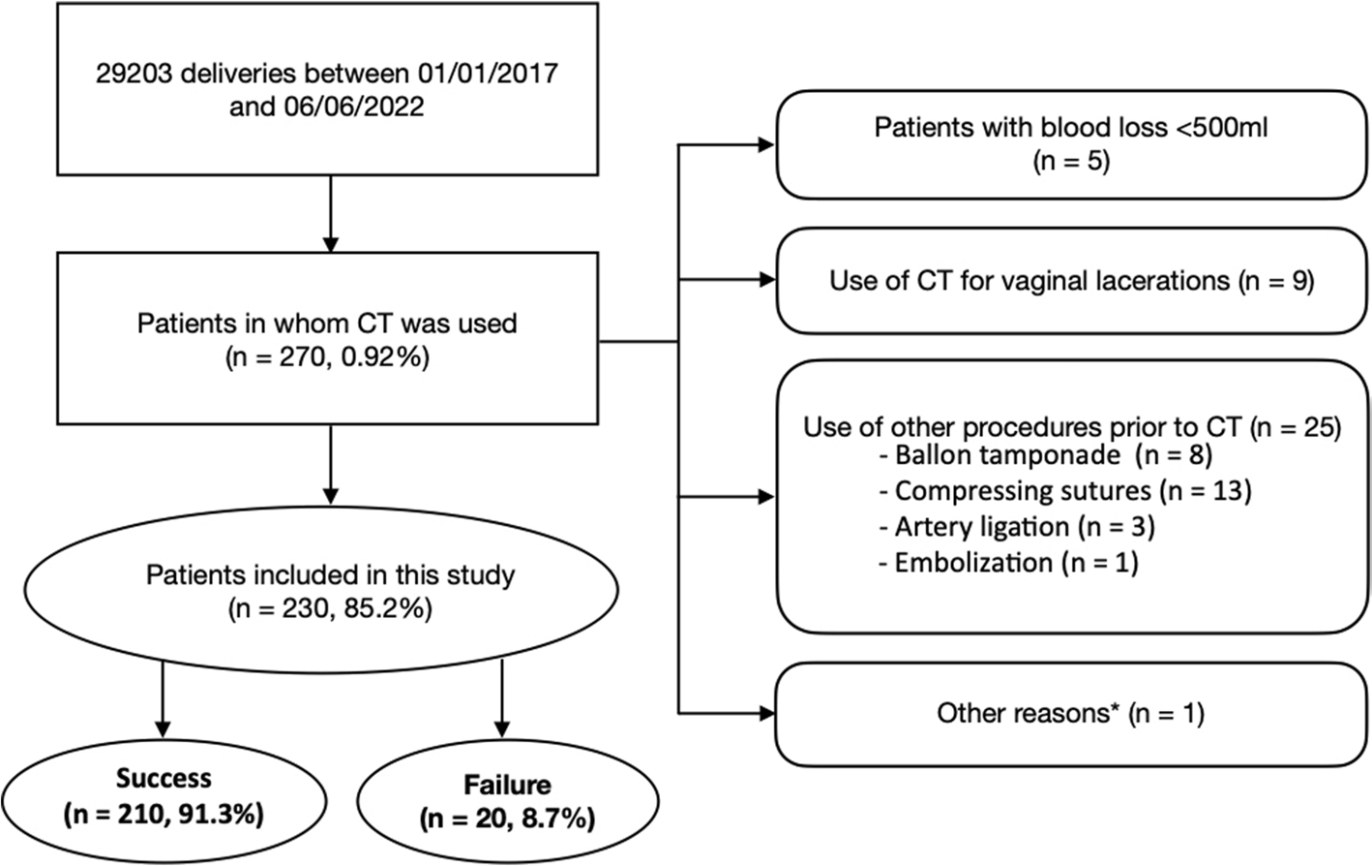
Flow chart based on inclusion and exclusion criteria additionally showing proportions of the CT treatment outcome. *other reason: in one patient CT was applied temporarily for few minutes because the patient developed heart failure during cesarean section and needed resuscitation; CT, chitosan-covered tamponade

Over a period of 5.5 years, a total of 270 women received a CT for PPH at the university perinatal center, representing 0.92% of all deliveries during this period. Finally, 230 women met the inclusion criteria and were included in this study (Fig. 1), where in 91.3% (210 patients) the use of CT effectively resulted in hemostasis. In the remaining 20 patients (8.7%) bleeding did not stop adequately, requiring further management.

### PPH characteristics

**Fig. 2:**
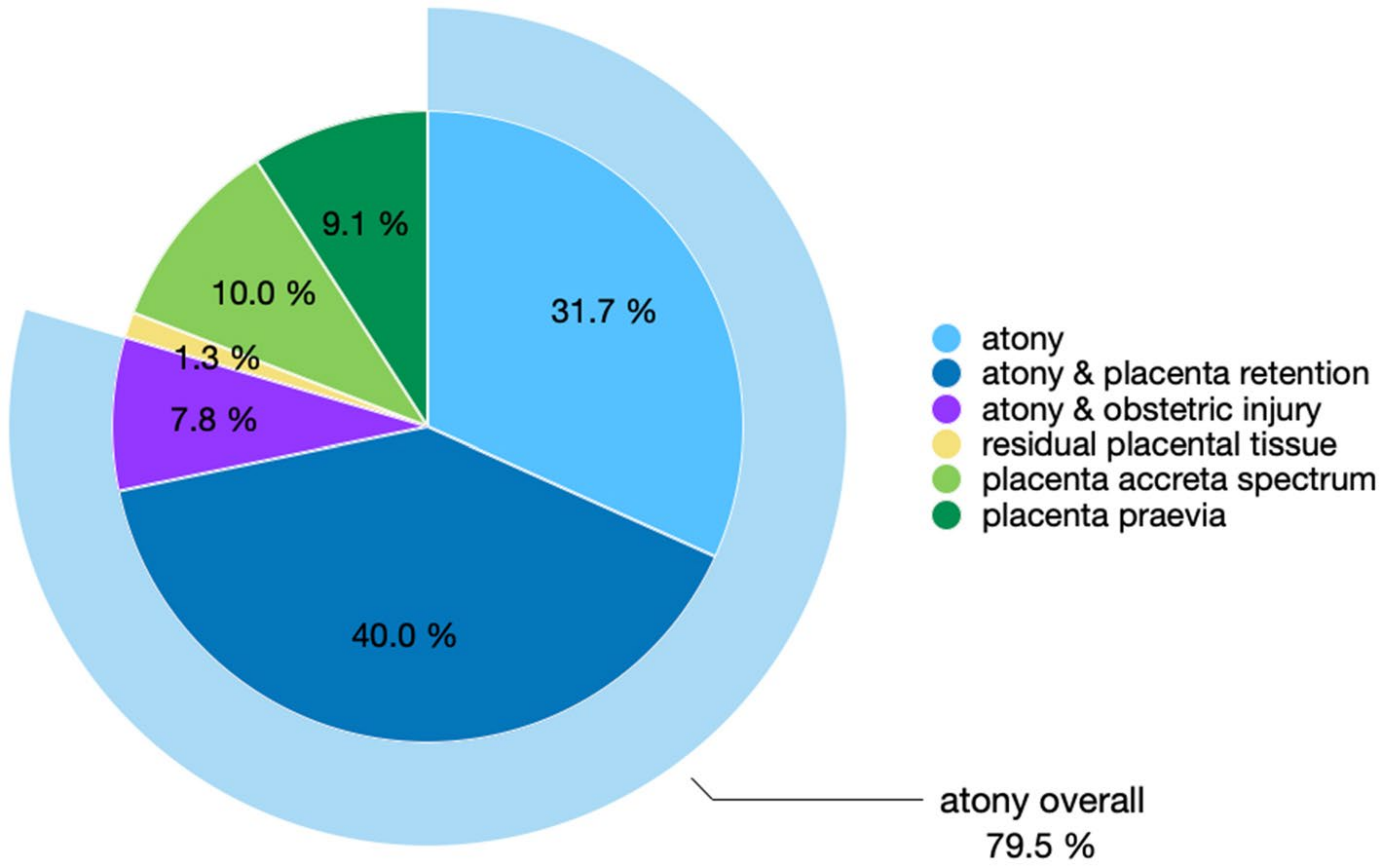
Relative frequencies of causes of PPH, based on a total of 230 patients with PPH

All patients had primary PPH, except for two patients where CT was successful, who presented with secondary PPH after 9 days and 3 weeks postpartum. PPH occurred mainly due to uterine atony (79.6% overall), including atony alone (31.7%), atony and placental retention (40.0%), and atony and obstetric lacerations (7.8%), followed by PAS (10.0%), placenta previa (9.1%), and residual placental tissue (1.3%, Fig. 2; Table 1). In 12 patients, placenta previa and PAS occurred combined.

**Table 1:** Details on clinical and laboratory parameters relevant for postpartum hemorrhage

	CT Success (*n*=210)	CT Failure (*n*=20)	All (*n*=230)
**Blood loss** (mL)			
**Prior to CT application**	1500 (1200-2000)	2000 (1575-2500)	1550 (1200-2000)
missing	-	6	6
**Total**	1500 (1200-2000)	3500 (1975-4875)	1700 (1200-2170)
**Causes of PPH**			
Atony	69 (32.9%)	4 (20.0%)	73 (31.7%)
Atony and placenta retention	89 (42.4%)	3 (15.0%)	92 (40.0%)
Atony and obstetric injury	15 (7.1%)	3 (15.0%)	18 (7.8%)
Residual placental tissue	3 (1.4%)	0 (0%)	3 (1.3%)
Placenta accreta spectrum	21 (10.0%)	2 (10.0%)	23 (10.0%)
Placenta previa	13 (6.2%)	8 (40.0%)	21 (9.1%)
**Hemoglobin level** (g/dL)			
**Prior to birth**	11.7 (10.7-12.5)	12.1 (11.1-13.0)	11.7 (10.7-12.6)
missing	30	6	36
**After birth**	6.8 (6.1-8.2)	6.2 (5.8-6.98)	6.8 (6.1-8.1)
missing	1	-	1
**Packs of gauzes**	1 (1-1)	1 (1-1.75)	1 (1-1)
**Insertion mode**			
Transvaginal	143 (68.1%)	10 (50.0%)	153 (66.5%)
Transabdominal	66 (31.4%)	10 (50.0%)	76 (33.0%)
Both	1 (0.5%)	0 (0%)	1 (0.4%)
**Duration of application **(hours)	24 (24-24)	21 (0.5-24)	24 (24-24)
missing	37	2	39
**Red blood cell concentrate**	85 (40.5%)	18 (90.0%)	103 (44.8%)
Packs	0 (0-2)	7 (2-8.25)	0 (0-2)
**Fresh frozen plasma**	46 (21.9%)	14 (70.0%)	60 (26.1%)
Packs	0 (0-0)	6 (0-9.25)	0 (0-1)
**Fibrinogen**	32 (15.2%)	12 (60.0%)	44 (19.1%)
**Uterotonics**	209 (99.5%)	20 (100%)	229 (99.6%)
Oxytocin	191 (91.0%)	16 (80.0%)	207 (90.0%)
Carbetocin	13 (6.2%)	3 (15.0%)	16 (7.0%)
Misoprostol	102 (48.6%)	10 (50.0%)	112 (48.7%)
Sulprostone	146 (69.5%)	19 (95.0%)	165 (71.7%)
**Tranexamic acid**	173 (82.4%)	20 (100%)	193 (83.9%)
**Admission to intensive care unit**	70 (33.3%)	16 (80.0%)	86 (37.4%)
**CRP** > 10 mg/dl	83 (39.5%)	15 (75.0%)	98 (42.6%)
missing	122	5	127
**Leuokocytes **> 17.000/µl	103 (49.0%)	12 (60.0%)	115 (50.0%)
missing	4	-	4
**Fever**	15 (7.1%)	1 (5.0%)	16 (7.0%)
**Hospital stay **(days)	4 (3-5)	7 (5-8.5)	4 (3-6)
missing	1	-	1

Continuous variables are presented as median and interquartile range. Categorical variables are presented as absolute and relative frequencies

**Fig. 3:**
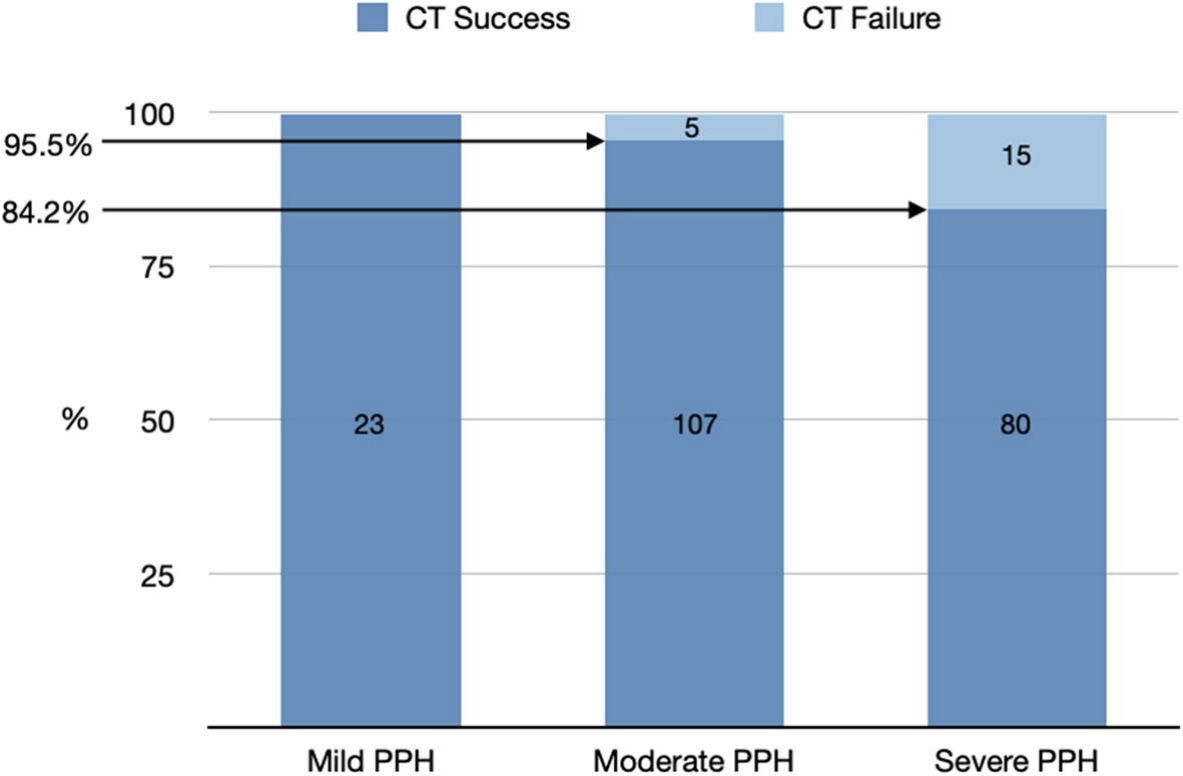
Success rates within the grades of postpartum hemorrhage (PPH); Grades according to the Royal College of Obstetricians and Gynecologists [21]; mild PPH: 500–999 mL, moderate PPH: 1000–1999 mL, severe PPH: ≥ 2000 mL

Patients with severe PPH were more likely to experience CT failure, as 75.0% of those with CT failure had severe and 25.0% had moderate PPH, compared to 38.1% and 51.0% of those with CT success, respectively. Therefore, the proportion of patients with successful CT treatment was 100.0% for mild, 95.5% for moderate, and 84.2% for severe PPH (Fig. 3). In the same lines, median total blood loss was lower in patients in whom CT was successful (1,500 mL (IQR 1,200-2,000) vs. 3,500 mL (IQR 1,975-4,875)). However, the median blood loss before CT application was not substantially different between the two groups (1,500mL (IQR 1,200-2,000) vs. 2000mL (IQR 1,575-2,500)) (Table 1).

### CT treatment of PPH

Depending on the mode of delivery, the CT was inserted either transvaginally or transabdominally. In one case, both insertion methods were used. The CT was always placed in the uterine cavity, and additionally in the vagina in four patients. Overall, 1 (80.9%) or 2 (14.2%) gauzes were used, with no differences with respect to the outcome.

Application of the CT was uncomplicated in all but three patients. Re-laparotomy became necessary twice because the repaired uterotomy at cesarean section was breached by transvaginal insertion of the CT and bleeding persisted. In one case, the gauze was sutured into the uterine wall during uterotomy closure. The duration of in-utero CT placement differed, as the gauze was often removed when CT failed and bleeding persisted.

Dislocation of the gauze into the vagina occurred in two patients shortly after insertion, once due to intense coughing after extubation, and once during the transfer of the patient from the operating table. The gauzes were reinserted without complications and with successful hemostasis in both cases.

### Pharmacological and supportive treatment of PPH

Almost all patients received oxytocin or carbetocin for bleeding prophylaxis. Approximately half of the patients were also administered misoprostol or sulprostone, which was applied substantially more often in those where CT failed. All patients with CT failure and 82.4% of patients with CT success received tranexamic acid.

Patients in whom CT failed received more red blood cell transfusions, more fresh frozen plasma, and more fibrinogen than patients with tamponade success. The majority of patients with successful CT outcome (59.5%) did not require any red blood cell transfusions, whereas 90.0% of patients with failed tamponade needed a blood transfusion, more than half of those patients received ≥ 7 units. They were admitted to the intensive care unit or the postoperative acute care unit more often (80.0% vs. 33.3%) and had a longer hospital stay than women with CT success (8.4 vs. 4.7 days, Table 1).

### Treatment upon CT failure

Interventions after the failure of CT ranged from sutures (*n* = 5), over artery ligation (*n* = 2), embolization (*n* = 1), balloon catheter (*n* = 3) to combined treatment (*n* = 6). Hysterectomy was decided upon as the last remaining option for stabilizing the patient’s condition and was performed a total of five times (Table 2). There were no maternal deaths in our cohort.

**Table 2:** Interventions in patients with CT failure, *n*=20

**Intervention after failure of CT**	**n**
**Sutures **	
B-Lynch or mattress sutures	3
Sutures at lower uterine segment	2
**Artery ligation**	2
**Emobilsation **	1
**Ballon catheter **	3
+ Artery ligation	2
+ Hysterectomy	1
+ B-Lynch sutures +/ artery ligation + hysterectomy	3
**Hysterectomy **	1
**Re-closure of breached uterotomy **	2

Of the five women who required hysterectomies after CT failed to adequately control bleeding, four had also undergone balloon therapy following CT. In only three patients where CT failed, the balloon was able to stop the bleeding, without further intervention. In two patients, bleeding persisted even after the ligation of one or both uterine arteries in addition to balloon therapy.

### Risk factors for CT failure

Patients in whom CT was successful were on average 2.5 years older (35.5 vs. 33), of higher parity (3 vs. 2), and of higher gestational age (40 vs. 38 weeks) (Table 3). The proportion of twin pregnancies was 11.0% (23/210) and 5.0% (1/20) among those with successful or failed CT, respectively, with additionally one triplet in each group. Intrauterine fetal deaths or late abortions occurred in a total of 10 patients, 7 of which were in the group with successful CT treatment. Two patients in the whole cohort had a known bleeding disorder (von Willebrand syndrome or idiopathic thrombocytopenia), four a HELLP syndrome – for all of them CT was successful. Ten patients had preeclampsia and one experienced eclampsia, eight of them had successful CT treatment.

**Table 3:** Patient characteristics, labor and delivery details and neonate outcomes

	CT Success (*n*=210)	CT Failure (*n*=20)	All (*n*=230)
**Maternal age **(years)	33 (27-37)	35.5 (31-37)	33 (28-37)
**BMI **(kg/m²)	23.4 (21-27.6)	25.2 (22.1-29.4)	23.5 (21-28)
missing	13	1	14
**Gravida**	2 (1-4)	3 (1.75-4.5)	2 (1-4)
**Para**	1 (1-2)	2 (1-3)	1 (1-3)
**Multiple pregnancy**	24 (11.4%)	2 (10.0%)	26 (11.3%)
Gemini	23 (10.9%)	1 (5.0%)	24 (10.4%)
Triplets	1 (0.5%)	1 (5.0%)	2 (0.9%)
missing	1	-	1
**Gestational week **(GW)			
Total	40 (38-41)	38 (35.75-40)	39 (37-41)
Delivery at ≥ 37 GW	158 (75.2%)	12 (60.0%)	170 (73.9%)
missing	1	-	1
**Anesthesia**			
General	100 (47.6%)	10 (50.0%)	110 (47.8%)
Peridural or spinal	66 (31.5%)	2 (10.0%)	68 (29.5%)
Peridural or spinal and General	44 (20.9%)	8 (40.0%)	52 (22.6%)
**Birth mode**			
Vaginal delivery	139 (66.2%)	7 (35.0%)	146 (63.5%)
Vacuum extraction	23 (11.0%)	3 (15.0%)	26 (11.3%)
Cesarean section	71 (33.8%)	13 (65.0%)	84 (36.5%)
**Lacerations of birth canal**	31 (22.0%)	1 (14.3%)	32 (21.9%)
**Placenta adherens**	119 (56.7%)	9 (45.0%)	128 (55.7%)
**Placenta accreta spectrum**	35 (16.7%)	8 (40.0%)	43 (18.7%)
Placenta accreta	30 (14.3%)	6 (30.0%)	36 (15.7%)
Placenta increta	4 (1.9%)	2 (10.0%)	6 (2.6%)
Placenta percreta	1 (0.5%)	0 (0%)	1 (0.4%)
**Placenta previa**	13 (6.2%)	8 (40.0%)	21 (9.1%)
**Manual removal of placental tissue**	135 (64.3%)	10 (50.0%)	145 (63.0%)
**Curettage**	97 (46.2%)	10 (50.0%)	107 (46.5%)
**Neonate weight **(grams)			
Total	3290 (2760-3760)	3000 (2367.5-3362.5)	3250 (2750-3710)
missing	4	1	5
Birth at ≥ 37 GW	3535 (3140-3900)	3232.5 (3005-3500)	3490 (3125-3857)
missing	1	-	1
**Neonate pH**	7.25 (7.2-7.29)	7.26 (7.2-7.3)	7.25 (7.2-7.29)
missing	11	3	14
**APGAR 5**	10 (9-10)	9 (9-10)	10 (9-10)
missing	12	3	15
**APGAR 10**	10 (9-10)	10 (9-10)	10 (9-10)
missing	12	3	15

Continuous variables are presented as median and interquartile range. Categorical variables are presented as absolute and relative frequencies

No remarkable differences were observed regarding baseline hemoglobin levels (median of 12.1 vs. 11.7 g/dL) prior to onset of labor, primary anesthesia type, gauze application mode (transvaginal or transabdominal), and curettage or manual removal of the placenta prior to CT insertion (Tables 1 and 3). General anesthesia was the most common form of primary anesthesia, administered to approximately half of the patients, followed by peridural and spinal anesthesia. In those patients where CT failed, general anesthesia became necessary after the bleed in almost all patients, resulting in 90.0% (18/20) receiving general anesthesia, in contrast to 68.5% (144/210) among those where CT was successful. In about half of the patients in both groups, curettage or manual removal of the placenta was performed.

In patients in whom CT failed, the interval between onset of bleeding and insertion of the CT was approximately 2 h in six women, which led to high blood loss prior to CT treatment. In another two patients, there was an unexpected presence of placenta increta, which was accompanied by fast and high blood loss. In patients with multiple pregnancies or atony of the lower segment of the uterus, the dilated lower uterine segment was not sufficiently packed (*n* = 5). In another two patients, there was severe tissue injury with bleeding from the uterine arteries, which was only diagnosed when hemorrhage persisted after CT insertion.

Mode of delivery was associated with CT failure, as cesarean deliveries were performed in 65.0% of patients with subsequent CT failure, and only in 33.8% with subsequent CT success. Lacerations of the cervix or birth canal were not associated with CT failure (Table 3).

 A comparison of the two groups showed considerable differences in placental implantation disorders. PAS was more frequent in women with CT failure (40.0% vs. 16.7% in women with successful treatment, Table 3). The most common condition in both groups was placenta accreta, followed by placenta increta. Placenta previa also occurred substantially more often among those where CT failed (40.0% vs. 6.2%). The placental bed or lower uterine segment was sutured in 10 (4.8%) and 3 (15.0%) patients with successful and failed treatment, respectively. Patients with PAS were comparable in BMI to those without PAS, but slightly older, presented more often with placenta previa, and had a lower neonatal weight (Table 4).

**Table 4:** Patient’s characteristics, stratified by the presence of placenta accreta spectrum

	**No placenta accreta spectrum** **(*n*=187)**	**Placenta accreta spectrum** **(*n*=43)**	**Total (*n*=230)**
**Maternal BMI in kg/m²**	23.7 (21.0, 27.6)	23.2 (21.1, 29.0)	23.5 (21.0, 27.9)
missing	12	2	14
**Maternal age **	31 (27, 36)	37 (33, 40)	33 (28, 37)
**Placenta previa**	9 (4.8%)	12 (27.9%)	21 (9.1%)
**Neonatal weight**	3345.0 (2786.5, 3795.0)	3027.5 (2372.5, 3351.3)	3250.0 (2750.0, 3710.0)
missing	4	1	5

**Fig. 4:**
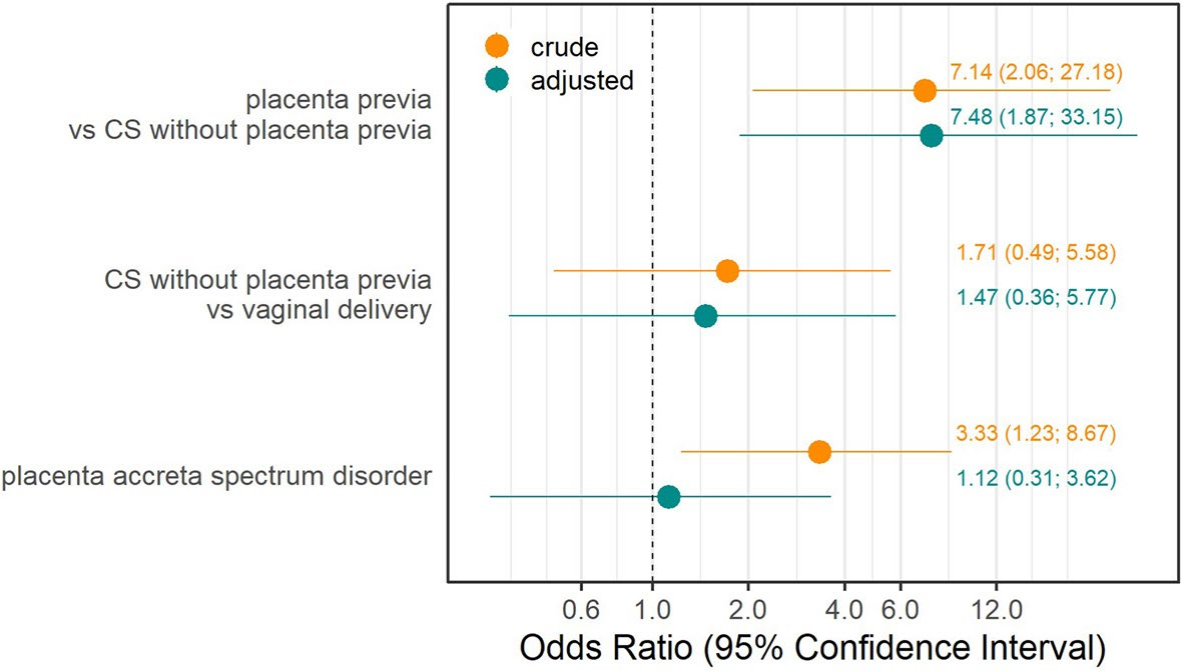
Binary logistic regression analysis for failure factors for CT failure. CS, cesarean section, PAS, placenta accreta spectrum disorder

**Fig. 5:**
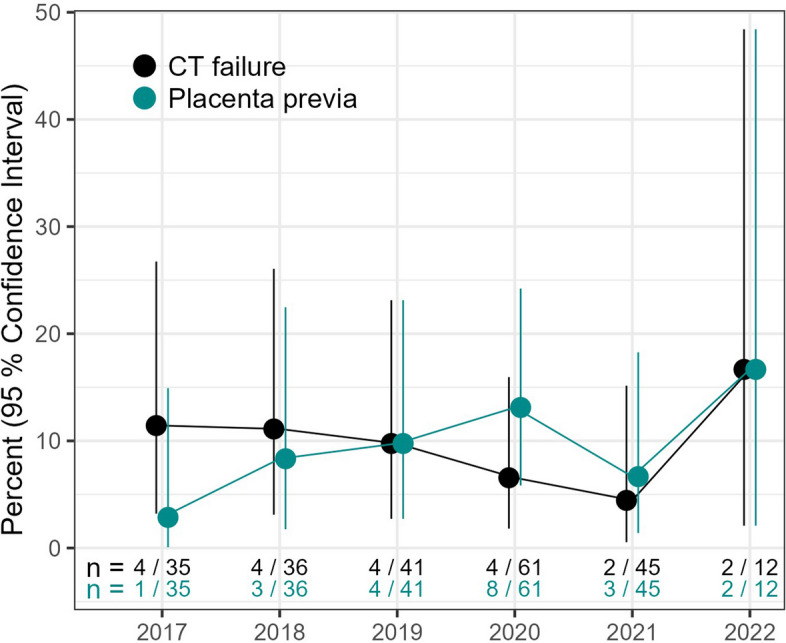
Development of CT failure and placenta previa over time

 After adjusting for confounders (BMI, maternal age, multiple pregnancies, neonatal weight, and PAS), the presence of placenta previa in cesarean sections increased the odds of CT treatment failure 7.5-fold compared to cesarean sections without placenta previa (OR = 7.48, 95% CI: 1.87–33.15). A cesarean section without placenta previa does not seem to pose a risk for CT failure, given the very wide confidence interval (OR = 1.47, 95% CI: 0.36–5.77). PAS did not have any influence on CT treatment failure (OR = 1.12, 95% CI 0.31–3.62) after adjusting for confounders (placenta previa, maternal age, increased BMI, and neonatal weight) (Fig. 4). Descriptively, during the study period from 2017 to 2022, we observed a slight downward trend in CT failures until 2021, but overall the numbers were relatively stable. There might also be a descriptive association between the proportion of CT failures and placenta previa cases per year, in line with the findings from the model (Fig. 5).

### Long term follow-up

At the end of the data collection in December 2023, the birth rate after the index birth was assessed in the whole cohort (*n* = 230). Of these, 22 patients with CT success and 1 patient with CT failure had a subsequent birth, all of which were uncomplicated with the exception of four PPH events, in which a CT was successfully applied again without complications.

## Discussion

### Summary of findings

Chitosan-covered tamponade has proven to be an effective method for hemostasis in PPH, with an overall success rate of 91.3%, offering a quick and straightforward treatment while reducing the need for more invasive interventions. This is the first analysis of risk factors for CT treatment failure. In our cohort of 230 women, 90.0% had moderate or severe PPH, and the most common reason for bleeding was uterine atony. The presence of placenta previa was the primary risk factor, increasing the odds of treatment failure 7.5-fold after adjusting for confounders. Cesarean sections did not seem to have an influence on treatment failure, while PAS did not relevantly increase the odds of persistent hemorrhage after CT, once confounders were considered. Other known risk factors for failed PPH therapy, such as maternal age, multiple pregnancies, curettage prior to insertion, pre-delivery anemia, the severity of blood loss prior to insertion of the intrauterine device, stillbirth, and neonatal macrosomia, did not relevantly influence the effectivity of CT in our cohort [25–28].

### Comparison to other studies

Previous studies have reported an association between cesarean delivery and failure of PPH management, particularly with intrauterine balloon tamponade [29], reporting an OR for treatment failure of up to 10.5 (95% CI 2.1–52.4) [30]. This may be due to scars in the uterine wall and larger wound areas compared to vaginal deliveries, leading to atony and increased bleeding. The underlying causes for performing a cesarean section, such as twin pregnancies or PAS, are themselves risk factors for PPH. In our cohort, 65.0% of women with failed CT treatment and 33.8% of women with successful treatment had cesarean deliveries. After adjustment for BMI, age, multiple pregnancies, PAS, and neonatal weight, cesarean section increased the odds of failure 1.4-fold compared to vaginal birth, albeit with a very wide confidence interval.

It should be emphasized that proper training and technique are important when inserting CT, particularly in centers with limited experience in its application. During CT insertion through the abdomen after cesarean section, it is crucial to avoid suturing the gauze into the uterine wall during uterotomy closure, which happened once in our cohort. For vaginal insertion, special care must be taken to prevent re-opening the uterotomy incision. This safety can be achieved by strictly inserting the gauze under ultrasound control or while the abdomen is still open in cesarean sections. Training in the correct use of CT is simple and fast, and our learning curve was steep, with complications in the application of CT only occurring early in the study.

Placenta previa is a well-known cause of PPH and remains a challenging obstetric condition because it is often accompanied by PAS and atony of the lower uterine segment with severe placental bed bleeding [31]. Currently, there is no gold standard device for the management of placenta previa bleeding. After adjusting for BMI, age, PAS, and neonatal weight, the presence of placenta previa significantly increased the odds of CT treatment failure 7.48-fold compared to cesarean sections without placenta previa. For CT to be effective, it must be in sufficient contact with the hemorrhaging tissue, which is complicated by the reduced contractility of the lower uterine segment in placenta previa, often requiring the use of more than one tamponade. In our cohort, CT was effective in 61.9% of patients with placenta previa, successfully treating 13 out of 21 patients by CT alone. CT treatment had to be combined mainly with sutures to stop bleeding in the remaining cases. In such cases, early intervention with the B-Lynch procedure or a transverse gathering seam or an intrauterine balloon is recommended [32]. We recommend the use of compression sutures before the application of CT, otherwise its removal may be difficult due to compression or suturing of the gauze.

An insufficient amount of gauze is a risk factor for treatment failure in multiple pregnancies. In our cohort, five women experienced failure due to insufficient packing of the uterine cavity with only one gauze. In such cases, combining intrauterine devices may be beneficial, as demonstrated in a triplet pregnancy with consecutive severe PPH after cesarean delivery, where CT and balloon tamponade were successfully used together [33]. There are no contraindications or disadvantages to using multiple gauzes to fill the large intrauterine space; in contrast, with intrauterine balloon therapy, the extrusion of the maximally filled balloon is a concern in up to 16% of cases [30].

There is very little RCT evidence of effectiveness of any intrauterine mechanical method in PPH treatment [34]. One recently published, very small randomized trial on a vacuum-induced device suggested benefits of suction tamponade over condom tamponade or uterine artery clamp [35]. RCTs on the effectiveness of CT treatment are still lacking.

The use of intrauterine balloon therapy in the presence of PAS and severe PPH is controversial [36]. Placental disorders are risk factors for balloon tamponade failure [37]. In our study, PAS disorders were more frequent in women with failed CT (40.0%) than in women with successful CT treatment (16.7%), highlighting the difficulty in successfully treating placenta increta and accreta bleeding. However, after adjusting for confounders such as placenta previa, maternal age, and neonatal weight, the OR for PAS was 1.1, indicating that PAS does not have a strong impact on CT treatment failure.

The association between placenta previa and PAS is well established. Obstetricians should be aware that women with a combination of these two risk factors for PPH as the underlying indications for cesarean section are at high risk of intrauterine therapy failure. However, CT as the initial second-line treatment could serve as a bridging method, particularly in cases of unexpected PAS, helping to stabilize the patient and gain time to prepare for alternative treatment options, such as segmental resection of the uterine wall, compression sutures, embolization, or ligation of the uterine arteries.

Prior to tamponade insertion, it is essential to ensure that any retained placental tissue is removed and any birth injuries are addressed. If the patient remains hemodynamically unstable despite these measures, immediate exclusion of tissue injury with arterial bleeding is necessary. In our study, two women required vascular ligation or hysterectomy after late diagnosed uterine artery bleeds.

The timing of CT treatment initiation is crucial for its success. Prolonged bleeding and substantial volume loss with development of coagulopathy prior to the insertion of intrauterine devices can necessitate more complex and invasive management strategies, and reduce CT efficacy [38]. In our study, 6 out of 20 women with CT failure experienced an average delay of two hours from bleeding onset to CT application compared to women where CT was successful, meaning that the tamponade was less effective in those patients with sudden high-flow bleeding (unexpected placenta accreta). It can be inferred that in them, there was not enough time for the interaction of the blood with the chitosan since the high flow did not allow it. Literature supports that longer delays before intrauterine balloon tamponade insertion result in poorer outcomes [39–41]. A French prospective cohort study of 226 women found that estimated blood loss prior to insertion of intrauterine balloon tamponade and subsequent coagulopathy was associated with therapy failure (OR 3.2 (95% CI 1.5–6.8) and OR 5.6 (95% CI 2.5–13.0), respectively) [42]. In addition, substantial early blood loss after balloon placement (> 200 mL at 10 min) is a prognostic factor for early balloon tamponade failure [43]. Against this background, early CT deployment should be considered in PPH management algorithms, alongside medical treatment, transforming the strictly vertical PPH algorithm to a more horizontal flow [42]. In stable patients with minor total blood loss, CT could be removed promptly, though concerns that the early use of intrauterine devices may lead to overuse must be acknowledged [9].

### Strengths and limitations

This study is the first to evaluate risk factors for the failure of non-balloon intrauterine therapy for the treatment of PPH. It is based on data collected both prospectively and retrospectively over 5.5 years in a registry of a large perinatal center. The births were carefully documented, resulting in minimal missing data.

The main limitation of this study is that it only reflects the clinical experience of a single perinatal center, rendering generalization of findings difficult. We cannot adequately account for changing personnel and increasing experience with CT use over the study period, potentially indicated by a small downward trend in CT failures from 2017 to 2021. Additionally, we did not compare the chitosan covered with a standard gauze tamponade, so the real effectiveness of the product could not be properly evaluated since the gauze itself produces hemostasis by compression and contact factor. Due to missing data, we could not fully investigate the time between onset of bleeding and tamponade insertion. However, our ongoing prospective trial will focus on accurately documenting time from PPH onset to treatment decision and tamponade insertion. As tamponade insertion decisions were made individually by the attending obstetrician, this might have introduced selection bias, as CT was perhaps only applied in cases where its success was more likely. However, the consistent application of a stepwise PPH management protocol potentially limited this bias.

## Conclusions

Chitosan-covered tamponade effectively treated postpartum hemorrhage in over 91% of patients. A primary risk factor for CT failure is placenta previa. Delayed CT insertion may also contribute to treatment failure. For patients with placenta previa and persistent bleeding, combining CT with compression sutures may be a viable treatment option. We recommend early use of CT in PPH management, as it poses no risks when applied by trained practitioners and may prevent the need for more invasive interventions. For patients requiring additional treatment, CT can serve as a temporary measure to reduce blood loss.

## Data Availability

Data will be provided by the corresponding author upon request.

## Abbreviations

BMI: Body mass index
CI: Confidence interval
CT: Chitosan-covered tamponade
HELLP: Hemolysis, Elevated Liver enzyme levels, and Low Platelet levels
IQRs: Interquartile ranges
IU: International units
mL: Milliliter
OR: Odds ratio
PAS: Placenta accreta spectrum (abnormally invasive placenta)
PPH: Postpartum hemorrhage
RCOG: Royal College of Obstetricians and Gynecologists
RCT: Randomized Controlled Trial
